# Misdirection due to early magnetoencephalographic presentation and management in Rasmussen encephalitis: a case report

**DOI:** 10.3389/fneur.2023.1261104

**Published:** 2023-11-30

**Authors:** Jinkun Han, Xiaotong Fan, Sichang Chen

**Affiliations:** Department of Neurosurgery, Xuanwu Hospital Capital Medical University, Beijing, China

**Keywords:** Rasmussen encephalitis, pediatric epilepsy, magnetoencephalography, hemisperectomy, electroencephalogram (EEG)

## Abstract

Rasmussen encephalitis is a rare and unexplained chronic brain hemispheric inflammatory disease. We report a case of epilepsy in which magnetoencephalography showed dipoles localized only in the operculum. Because the patient’s clinical presentation and examination findings did not meet the diagnostic criteria for Rasmussen encephalitis, he underwent cortical electroencephalogram (ECoG) record and limited resection surgery. However, the seizures were not relieved after surgery, and imaging findings showed significant features of hemisphere atrophy. This young male patient was eventually diagnosed with Rasmussen encephalitis and the seizures was completely vanished following hemispherectomy. His data can provide a reference for the early identification of this devastating disease.

## 1 Background

Rasmussen encephalitis is a rare brain disorder that can lead to epilepsy and occurs mostly in children. Its mean age of onset is 6 years. Reportedly, approximately 2.4 out of every 100,000 people in Germany suffer from this disease (1). These patients often have focal epilepsy, progressive hemiplegia, and atrophy of one hemisphere (2, 3). Histopathological examination shows infiltration of T cells and microglia as prominent features. This case presents a tortuous course of diagnosis and treatment of Rasmussen encephalitis.

## 2 Case details

**Figure 1:**
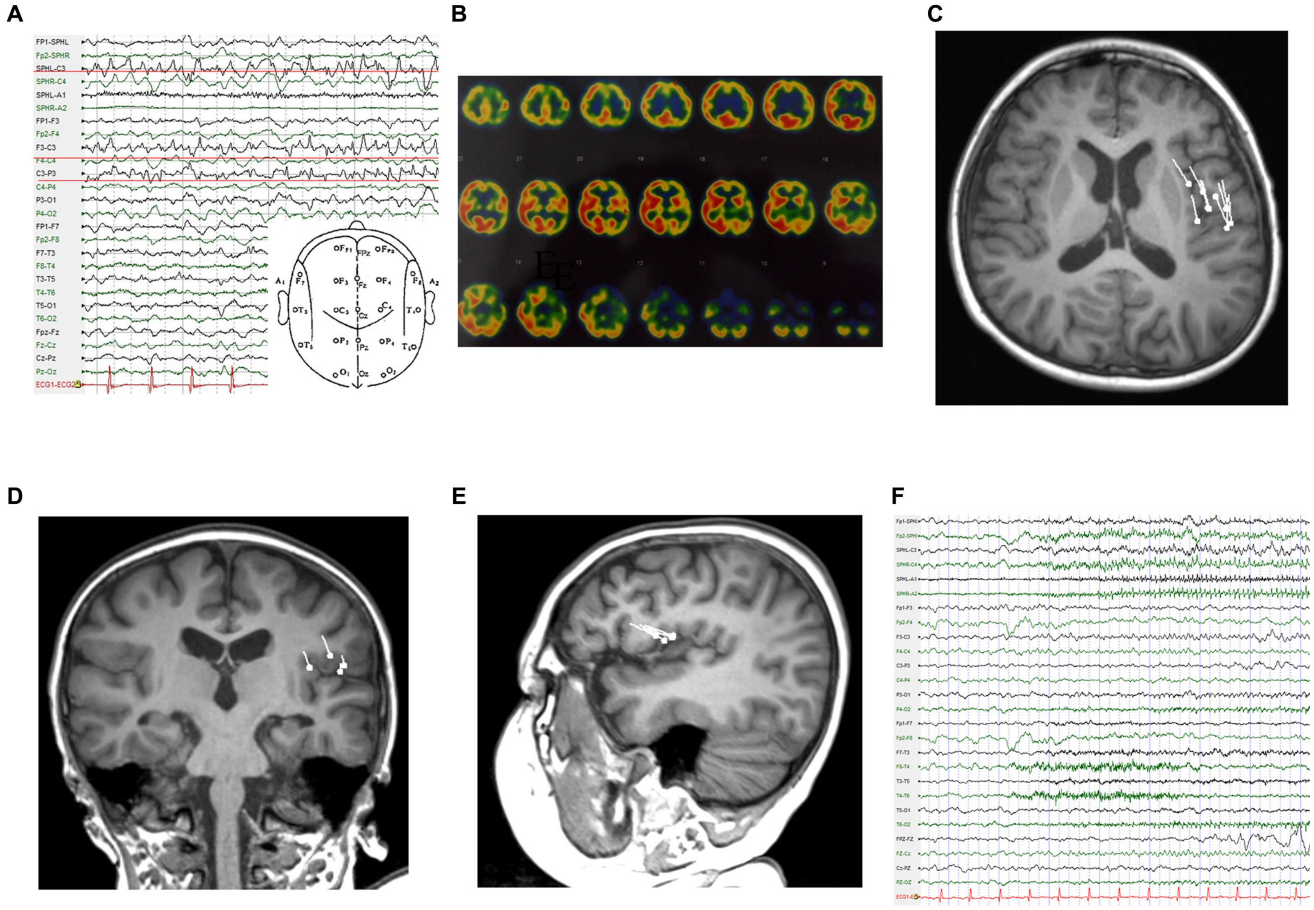
Examinations in the first admission. **(A)** Interictal EEG. **(B)** SPECT reveals markedly hypointense areas. **(C-E)** Dipoles are concentrated in the left operculum on magnetoencephalography (C: T1 axial, D: T1 coronal, E: T1 sagittal). **(F)** Ictal EEG.

The patient was a 4-year-old boy who was admitted to the department of pediatrics with a 6-month history of episodic vomiting and convulsions. During seizures, he was unable to speak and showed facial twitching and head rotation, followed by rhythmic twitching of the right upper and lower extremities. These symptoms lasted for 1–2 min and then resolved. After remission, he had slurred speech and sometimes even an inability to pronounce words, along with weakness of the right limbs, which lasted for approximately 10 min. He had recently had 7–8 attacks per day. The patient took sodium valproate, oxcarbazepine, and clonazepam before admission. He had no previous history of trauma or surgery, and no history of febrile seizures, no abnormal events had occurred in the perinatal period. During the interictal period, the patient had slightly poor language function, normal muscle strength of the extremities, and negative pathological signs. Electroencephalography (EEG) after admission showed a spike rhythm in the left frontal and left central areas during the interictal period (Figure 1A). The ictal EEG showed rhythmic discharges in the left central area (Figure 1F). On single-photon emission computed tomography (SPECT) interictally, regional blood perfusion was decreased in the left frontal, parietal, temporal, and occipital cortex compared with the corresponding regions on the contralateral side, and poor regional blood perfusion was observed in the bilateral anterior temporal lobes (Figure 1B). Notably, the dipoles were densely distributed in the left inferior frontal lobe and left insula in the magnetoencephalogram performed for surgical evaluation (Figures 1C–E).

**Figure 2:**
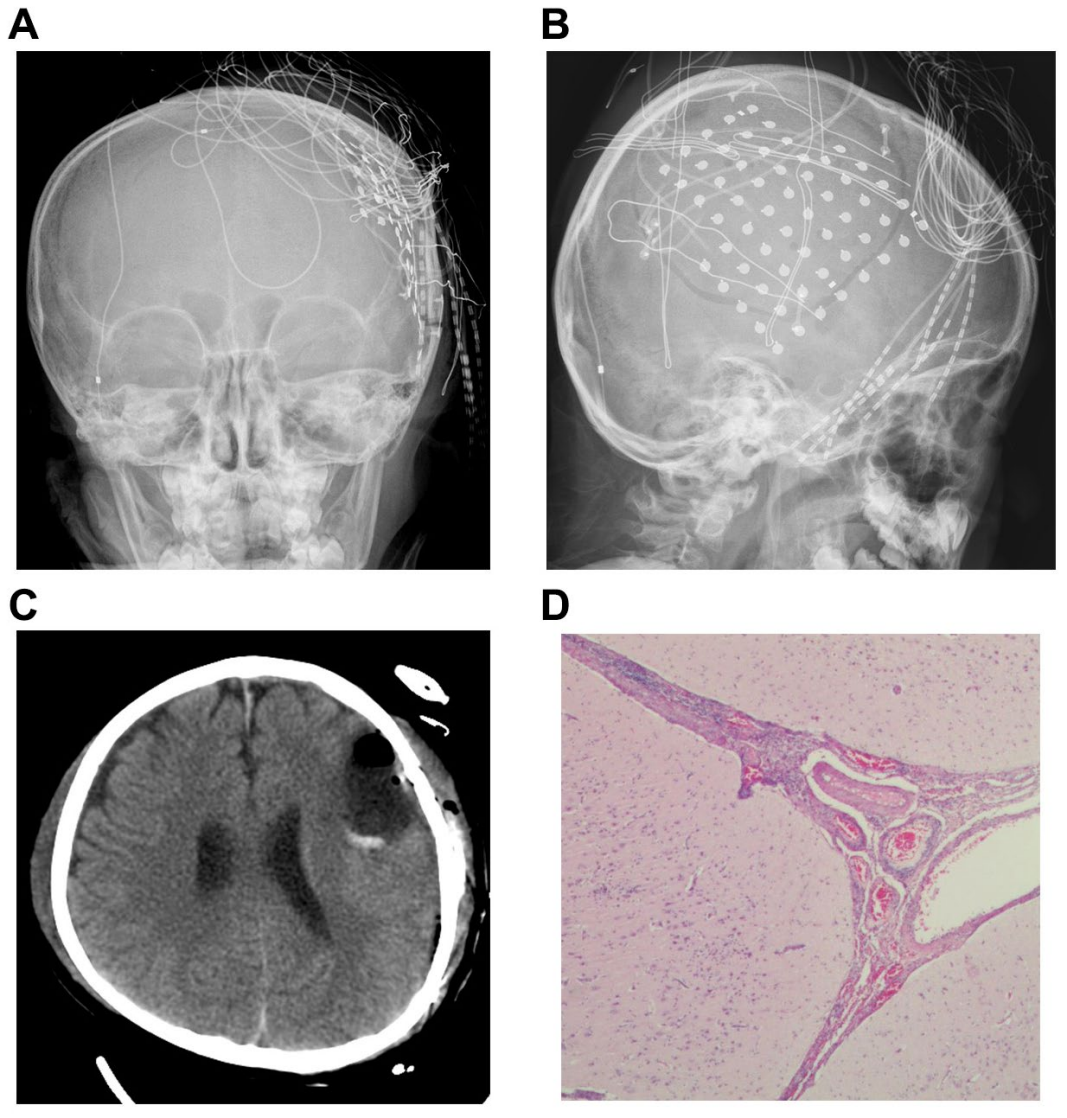
Imaging and pathology. **(A,B)** Computed radiography demonstrates the location where the cortical electroencephalogram was recorded. **(C)** Re-examination after resection of the epileptic focus. **(D)** Pathological findings after surgery led to a diagnosis of meningoencephalitis (Hematoxylin and eosin (H&E) stained images).

According to the clinical symptoms, EEG, MRI, magnetoencephalography (MEG), and SPECT, we inferred that the left inferior frontal gyrus played an important role in this patient’s epileptic seizures, and an electrocorticography (ECoG) electrode was surgically implanted under the dura mater to cover the superior temporal gyrus, middle frontal gyrus, inferior frontal gyrus, lateral fissure, central area, and parietal lobe (Figures 2A,B). We determined the locations of the epileptic foci in relation to eloquent areas by cortical electrical stimulation, and we decided that the next surgical plan should be resection of the epileptic foci in the left middle and posterior frontal and left parietal lobes (Figure 2C). No abnormal EEG signals were detected in the cortex around the surgical area during the operation, and no seizures were clinically observed during the patient’s hospitalization after the operation. The pathological conclusion was that the lesion was frontal, and the patient was diagnosed with meningoencephalitis (Figure 2D).

However, the patient presented seizures again 16 days after resection surgery, and his symptoms were essentially the same as those before surgery, but the frequency was reduced. Six months later, he was readmitted to the pediatrics department for seizure assessment. At this time, physical examination revealed impaired language and cognitive function, along with decreased muscle strength on the right side of the body and slightly increased tone. Given the patient’s focal epilepsy that did not respond to local resection, combined with the postoperative pathological findings of meningoencephalitis, a suspicion of Rasmussen encephalitis began to develop. However, since the patient’s antiepileptic drug doses continued to increase and his intelligence did not decline significantly, the patient’s families rejected surgery including extended lesionectomy or hemispherotomy, and the boy was discharged.

**Figure 3:**
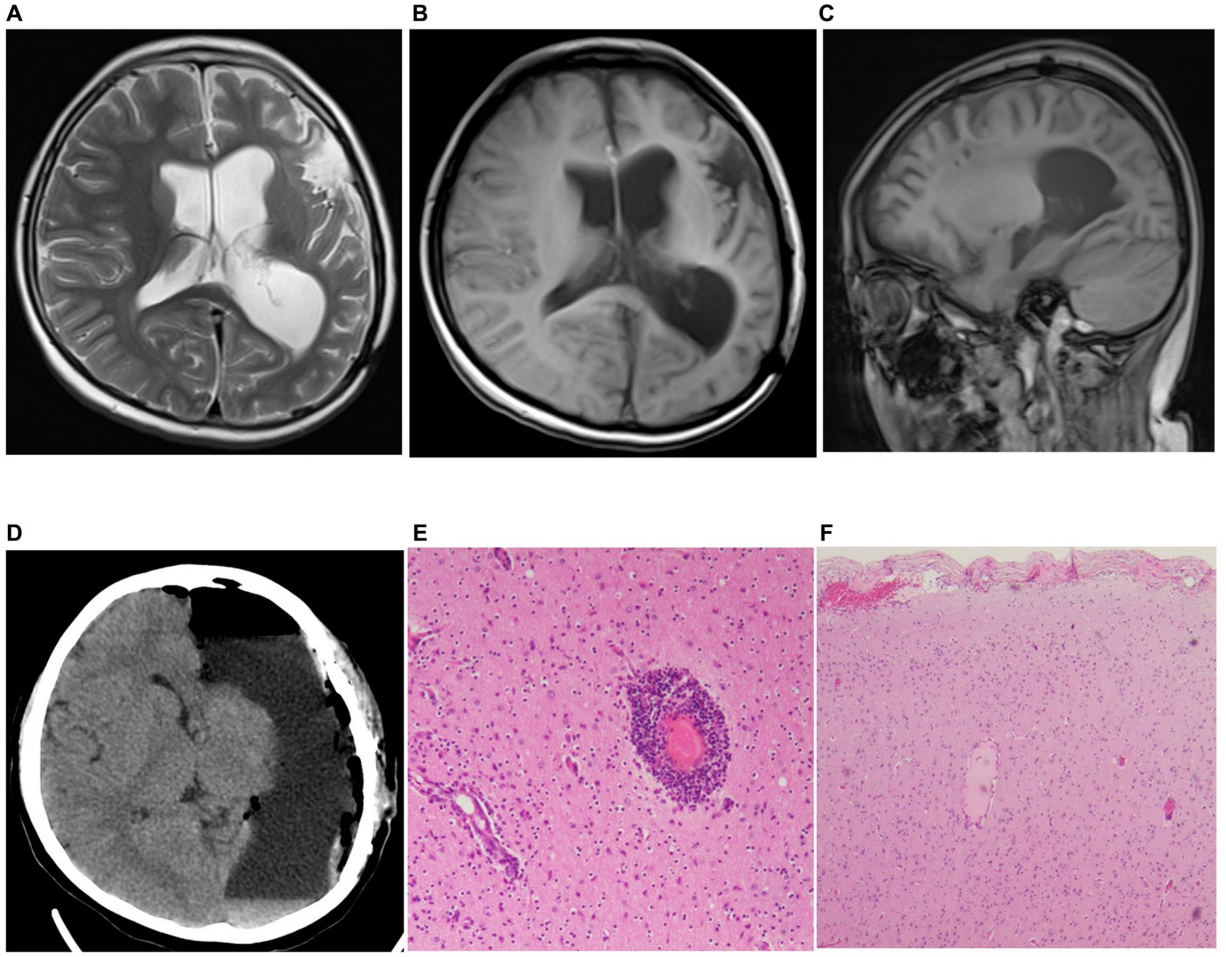
Preoperative MRI and postoperative pathology results from last surgery. **(A-C)** MRI results (A: T2 axial, B: T1 axial, C: T1 sagittal) showed marked atrophy of the patient’s brain tissue (including head of left caudate in T2 axial). **(D)** Results of reexamination after cerebral hemispherectomy. **(E,F)** Postoperative pathological findings from the second resection surgery met the diagnostic criteria for Rasmussen encephalitis (H&E stained images).

Two years later, the patient presented with frequent seizures and status epilepticus. He was readmitted to the pediatrics department. MRI data showed atrophic findings in the left frontotemporoparietal region and left caudate head (Figures 3A–C). PET-CT data showed areas of severely decreased radioactive tracer uptake in the left frontal, parietal, temporal, and occipital lobes. In addition, although the patient’s intelligence was the same as that of his peers before the onset of the disease, his mental development fell behind after the onset of the disease (even after surgery). Considering the persistent focal epilepsy symptoms and cortical atrophy of one cerebral hemisphere, the patient was ultimately diagnosed with Rasmussen encephalitis according to the European consensus diagnostic criteria (4) and was subsequently transferred to the neurosurgery department for hemispherectomy (Figure 3D). The second pathological examination revealed infiltration of microglia and lymphocytes, which is typical phynotype of Rasmussen encephalitis. Within 7 years after surgery, the patient was totally seizure-free (ILAE 1) (Figures 3E,3F).

## 3 Discussion

Rasmussen encephalitis has three stages: a prodromal phase of low-frequency seizures and mild hemiplegia; an acute phase of frequent seizures, often accompanied by progressive hemiplegia, hemianopia, and cognitive impairment following damage to the dominant hemisphere; and a sequela phase of persistent neurological impairment and persistent seizures. In this case, the patient was treated early after the onset of epilepsy. Even at this point, the results of ECoG and MEG did not support widespread lesions in the affected hemisphere. We were able to identify a trace of Rasmussen’s encephalitis from the patient’s preoperative examination and clinical presentation. The patient was at the prime age for the development of Rasmussen encephalitis. The patient’s symptoms were focal seizures and a feeling of weakness in the limbs during the interictal intervals. In addition, we found a decrease in blood perfusion in the lobes of one hemisphere. Although these pieces of evidence are indirect, they warrant diligent monitoring.

In the early stages of Rasmussen encephalitis, patients usually present with mild hemiplegia and mild seizures. The results may not be typical at this time. During the preoperative evaluation of this patient, scalp EEG and MEG provided valuable evidence that the extent of the epileptic foci was relatively limited, and these two techniques yielded concordant results. In particular, MEG clearly showed that dipoles were present in the parietal operculum and frontal operculum. This undoubtedly increases the difficulty of confirming the diagnosis. As a localization method, MEG is very reliable in some cases, especially when the dipoles are relatively concentrated (5). In this case, both MEG localization and cortical EEG localization were accurate, but Rasmussen encephalitis is a progressive disease, and early local resection did not prevent disease progression.

To our knowledge, no similar MEG findings in the early stages of Rasmussen encephalitis have been reported. The significance of studying this case is that similar MEG findings should not be a possible reason to rule out Rasmussen encephalitis, especially when other evidence can be used to indicate Rasmussen encephalitis. Moreover, such MEG findings can even provide a basis for the staging of Rasmussen encephalitis, that is, early or prodromal manifestations. The course of treatment in this patient may also suggest that for very frequent focal motor seizures, Rasmussen encephalitis may be present even in the absence of typical magnetic resonance findings. Megan has reported that some patients with Rasmussen encephalitis have no evidence of early magnetic resonance findings, such as cortical atrophy, which warrants a high suspicion of this epileptogenic disorder (6). In particular, the progression of Rasmussen encephalitis may be somewhat prolonged by the use of multiple novel antiepileptic drugs, but pharmacological treatment alone does not change the final outcome. Local excision surgery may have short-term effects in early-stage patients with Rasmussen encephalitis but does not alter long-term outcomes and instead delays the optimal window of treatment for patients. Definite diagnosis of early Rasmussen encephalitis in patients may allow surgeons to help them achieve a better prognosis by performing hemispherectomy earlier (7).

## 4 Conclusion

Early diagnosis of Rasmussen encephalitis promotes a favorable prognosis. We provide an interesting magnetoencephalographic presentation of an early Re encephalitis，which may reflect in multifocal/extensive pathologies, multiple epileptogenic networks may have different threshold of electrophysiological expression that can mislead to consider the one with highest epileptogenicity as the sole area responsible for seizure onset, warrants further investigation.

## Data availability statement

The original contributions presented in the study are included in the article/supplementary material, further inquiries can be directed to the corresponding author.

## Ethics statement

Ethical review and approval was not required for the study on human participants in accordance with the local legislation and institutional requirements. Written informed consent from the patients/participants or patients/participants' legal guardian/next of kin was not required to participate in this study in accordance with the national legislation and the institutional requirements. Written informed consent was obtained from the minor(s)' legal guardian/next of kin for the publication of any potentially identifiable images or data included in this article.

## Author contributions

JH: Formal analysis, Investigation, Methodology, Writing – original draft. XF: Conceptualization, Data curation, Writing – review & editing. SC: Resources, Supervision, Validation, Writing – review & editing.
